# A case report of isolated right ventricular noncompaction with mutation of *ACVRL1*: a new cause of noncompaction of ventricular myocardium?

**DOI:** 10.1186/s12872-023-03132-y

**Published:** 2023-04-29

**Authors:** Bo Yu, Kun Shi, Yang Wen, Yanfeng Yang

**Affiliations:** Chengdu Women’s and Children’s Central Hospital, School of Medicine, University of Electronic Science and Technology of China, Chengdu, 611731 China

**Keywords:** Isolated right ventricular noncompaction, Noncompaction of ventricular myocardium, *ACVRL1*, Case report

## Abstract

**Background:**

Noncompaction of ventricular myocardium(NVM) is a rare kind of cardiomyopathy associated with genetic mutations and nongenetic factors, among which the isolated right ventricular noncompaction (iRVNC) is the most rare type. *ACVRL1* is the pathogenic gene of type 2 hereditary hemorrhagic telangiectasia (HHT2), and there’s no NVM reported to be associated with *ACVRL1* mutation.

**Case presentation:**

This is a rare case diagnosed as iRVNC and pulmonary hypertention with *ACVRL1* mutation detected.

**Conclusion:**

iRVNC in this case may be due to *ACVRL1* mutation, secondary to pulmonary hypertention and right ventricular failure caused by *ACVRL1* mutation, or they happened in the same case coincidently.

**Supplementary Information:**

The online version contains supplementary material available at 10.1186/s12872-023-03132-y.

## Backgroud

Noncompaction of ventricular myocardium(NVM) is a rare sort of cardiomyopathy, among which isolated right ventricular noncompaction(iRVNC) is the most rare type [1, 2]. It’s an unclassified cardiomyopathy associated with genetic mutations and nongenetic factors [3, 4]. Mutations of related genes, for example, some sarcomeric protein genes *MYH7*, *MYBPC3*, *JUP* and pathogenic genes of Barth syndrome *TAZ* could lead to NVM [5, 6]. Activin receptor-like kinase 1 gene (*ACVRL1*) is the pathogenic gene of hereditary hemorrhagic telangiectasia type 2 (HHT2), which leads to arteriovenous fistula or proliferation of vascular wall, occlusion and reconstruction of pulmonary arterioles, and could eventually cause pulmonary hypertension [7–10]. To date, no NVM was reported to be associated with *ACVRL1* mutation.

Here, we report a 1 year and eleven-month old male child diagnosed as iRVNC and pulmonary hypertention, with *ACVRL1* mutation was detected.

## Case presentation

A male child, 1 year and eleven-month old, was hospitalized for "coughing for 1 week". About past medical history, the child had growth retardation and decreased activity. He had a small amount of epistaxis 2–3 times in the past, and his mother and grandmother from his mother’s side had repeated epistaxis when they were young similarly.

Physical examination on admission: Weight 10.5 kg(reference range:8.8 kg ~ 12.7 kg), Height 76.0 cm(reference range:77 cm ~ 88 cm), body mass index(BMI) 18.18 kg/m^2^(reference range 13.8 ~ 19.0 kg/m^2^), T 36.5℃, his pulse was recorded as 110beats/min, blood pressure (BP) as 87/49 mmHg and percutaneous oxygen saturation(SPO_2_) 96%. Clear mind, good response, ruddy lips, mild inspiratory three concave sign, pharyngeal congestion, enlargement of tonsil I degree and no secretion. Both lungs sounded rough and symmetrical and a small amount of rales could be heard; II/VI murmur in the systolic phase could be heard in the left sternal margin 4–5 intercostals. The abdomen was soft, and the liver, whose texture is medium, was 3 cm below the costal margin and 4 cm below the xiphoid process, and the spleen was not touched. The extremities were warm, no pestle strong fingers and toes.

**Fig. 1:**
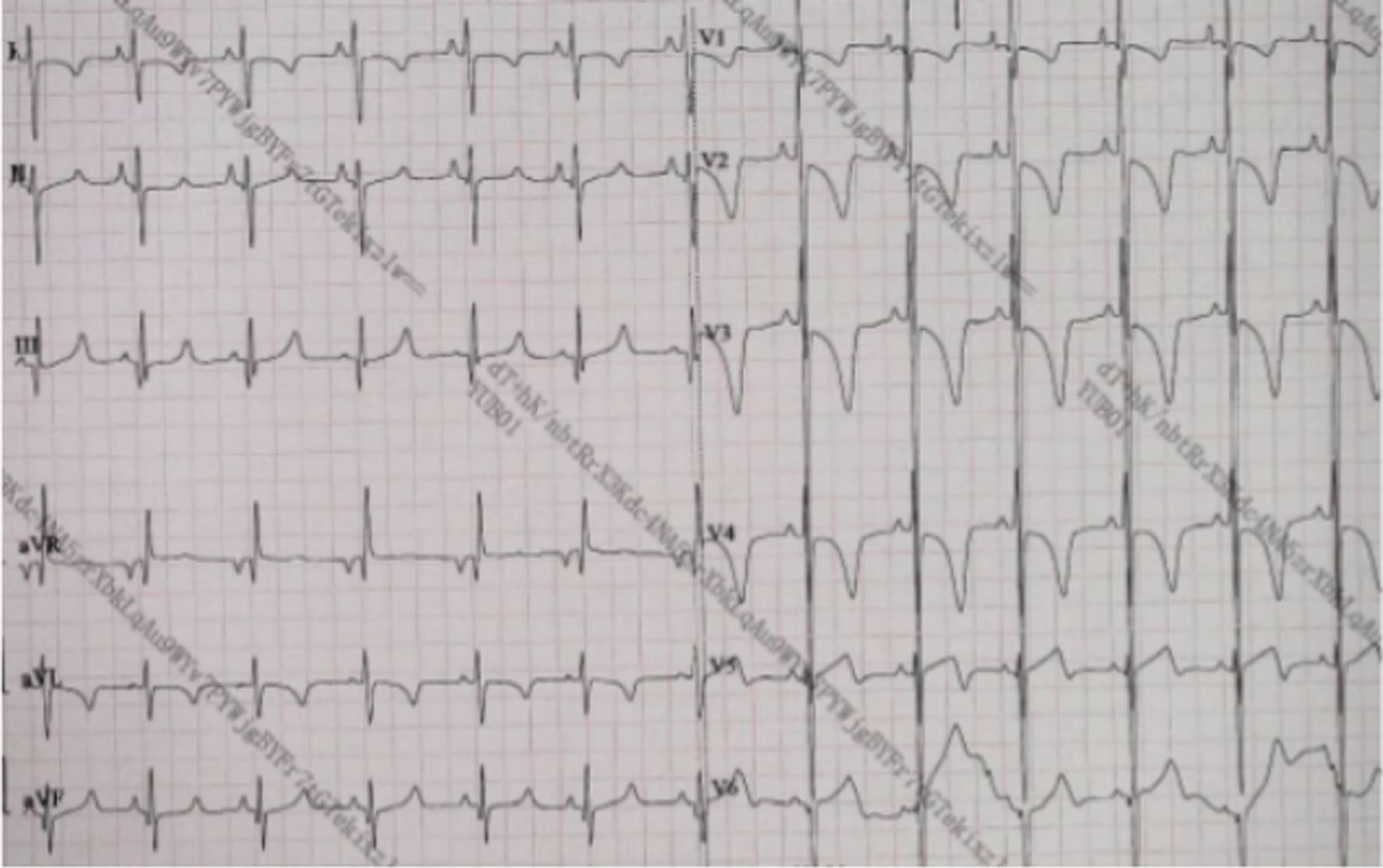
ECG revealed enlarged right atrium and right ventricle and ST-T changes

**Fig. 2:**
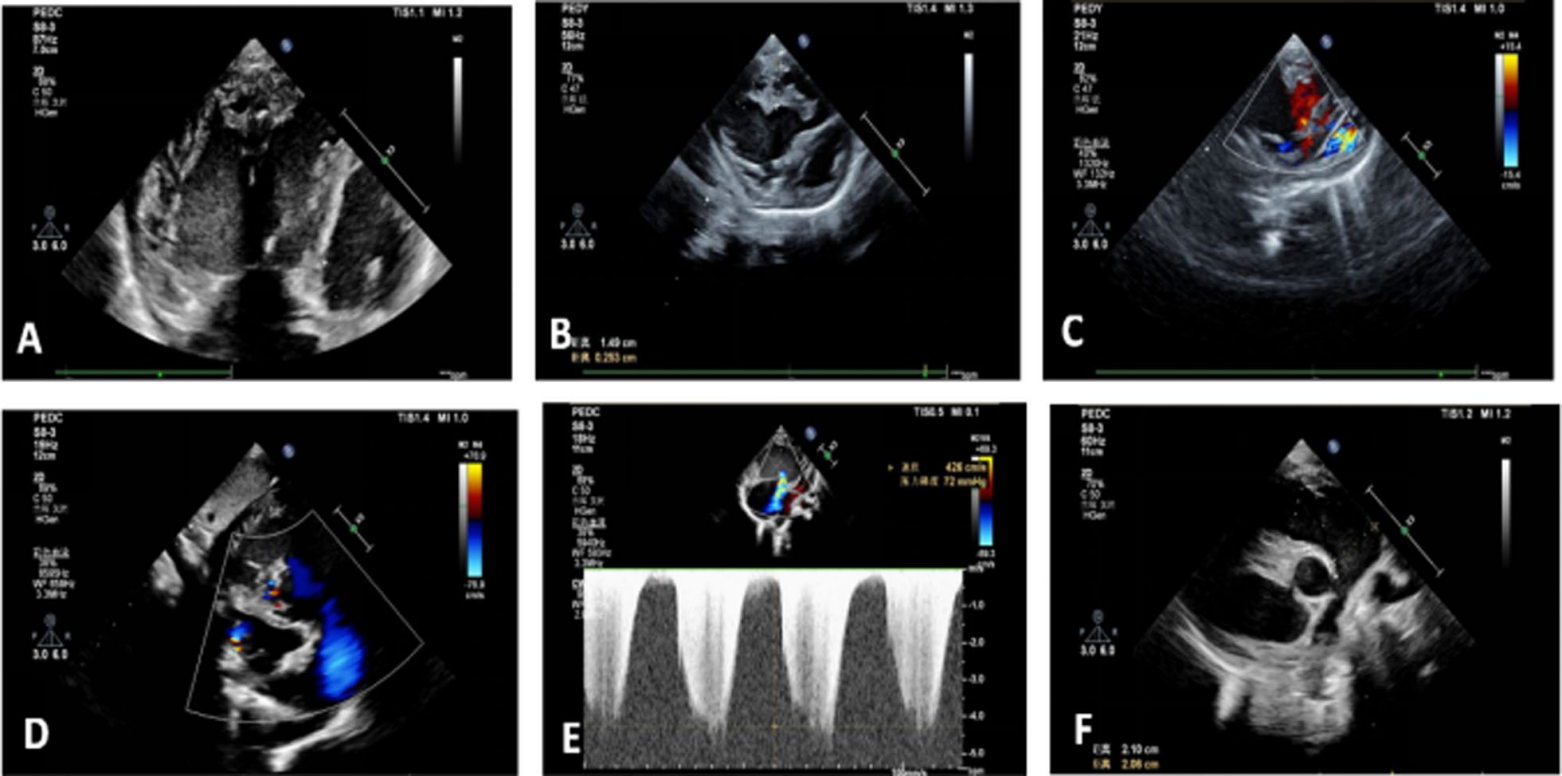
**a **Significantly enlargement of right ventricle, right atrium, right ventricular diameter 3.11 cm (reference range:0.90 cm ~ 1.99 cm), right atrium 3.82 cm*4.11 cm(reference dimeter:2.19 cm ~ 2.41 cm). **b **Non-compact layer composed of abundant trabecular muscle and intertrabecular recesses, and the ratio of non-compact layer to compact layer was 5.89:1(reference range: ≤ 2.0). **c**,** d **Low-speed blood flow among the recesses. **e **Tricuspid regurgitation (regurgitation velocity 4.26 m/s). **f **The diameter of pulmonary artery were widened, main pulmonary artery diameter:2.06 cm(reference range:0.95 cm ~ 1.58 cm)

Auxiliary examination: blood routine examination and high-sensitivity C-reactive protein(hs-CRP): total leukocyte count: 7.05 × 10^9^ / L, neutrophil percentage: 29.3%, lymphocyte percentage: 59.4%, hemoglobin determination: 93 g / L, total platelet count: 152 × 10^9^ / L, hs-CRP: < 1.0 mg/l; N-terminal pro NT-proBNPbrain natriuretic peptide(NT-proBNP) 2664.7 pg/ml (referamce range:0-300 pg/ml). No abnormality was observed in urine, feces, biochemical examination, sceening of hematuria, thyroid function and autoantibodies. Electrocardiogram revealed enlarged right atrium and right ventricle and ST-T changes (Fig. 1). Cardiac ultrasound color Doppler: significant enlargement of right ventricle and smaller left ventricle (Fig. 2a), non-compact layer composed of abundant trabecular muscle and intertrabecular recesses, and the ratio of non-compact layer to compact layer was 5.89:1(Reference range: ≤ 2.0) (Fig. 2b), low-speed blood flow among the recesses (Fig. 2c,d), moderate-severe tricuspid regurgitation (regurgitation velocity 4.26 m/s) (Fig. 2e), the diameter of right ventricular outflow tract and pulmonary artery were widened (Fig. 2f). Computed tomography angiography(CTA): enlargement of the right atrium and ventricle, smaller left ventricle, right ventricular noncompaction, widening of the main pulmonary artery and branches, a small amount of pericardial effusion and extensive patchy shadows in both lungs. No clumpy, nodular, or cystomatous lesions that could be enhanced, either no thickening or tortuosity of pulmonary veins could be seen on CT.

**Fig. 3:**
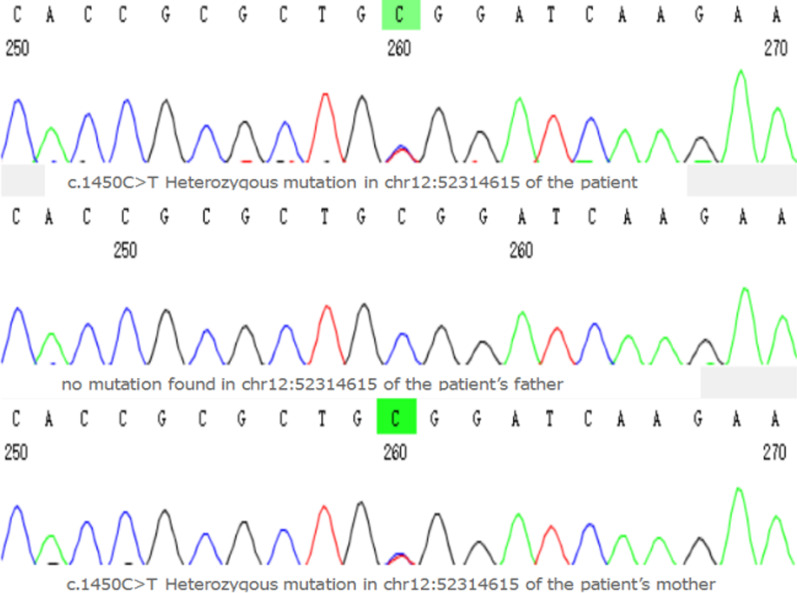
Pyrosequencing profiles of the three genotypes of the c.1450C > T(chr12:52,314,615) *ACVRL1* mutation were identified.The patient and his mother carried this same genetic mutation

We diagnosed the boy with: 1. iRVNC 2. Pulmonary hypertension 3. Pneumonia. After 10 days of treatment with piperacillin sulbactam anti-infection, furosemide and spironolactone diuresis, digoxin cardiomycin, metoprolol to reduce sympathetic excitation and aspirin anti-platelet, his pneumonia was cured and cardiac function was improved. After 2 weeks, the whole exon gene assay indicated activin A receptor-like 1 gene (*ACVRL1*) mutation of c.1450C > T:p.R484W, which was inherited from his mother confirmed by first-generation sequencing (Fig. 3). His mother's cardiac ultrasound showed left ventricle apex noncompaction. The prediction of protein structure model suggested this point mutation slightly affected the conformation of *ACVRL1* protein.

**Fig. 4:**
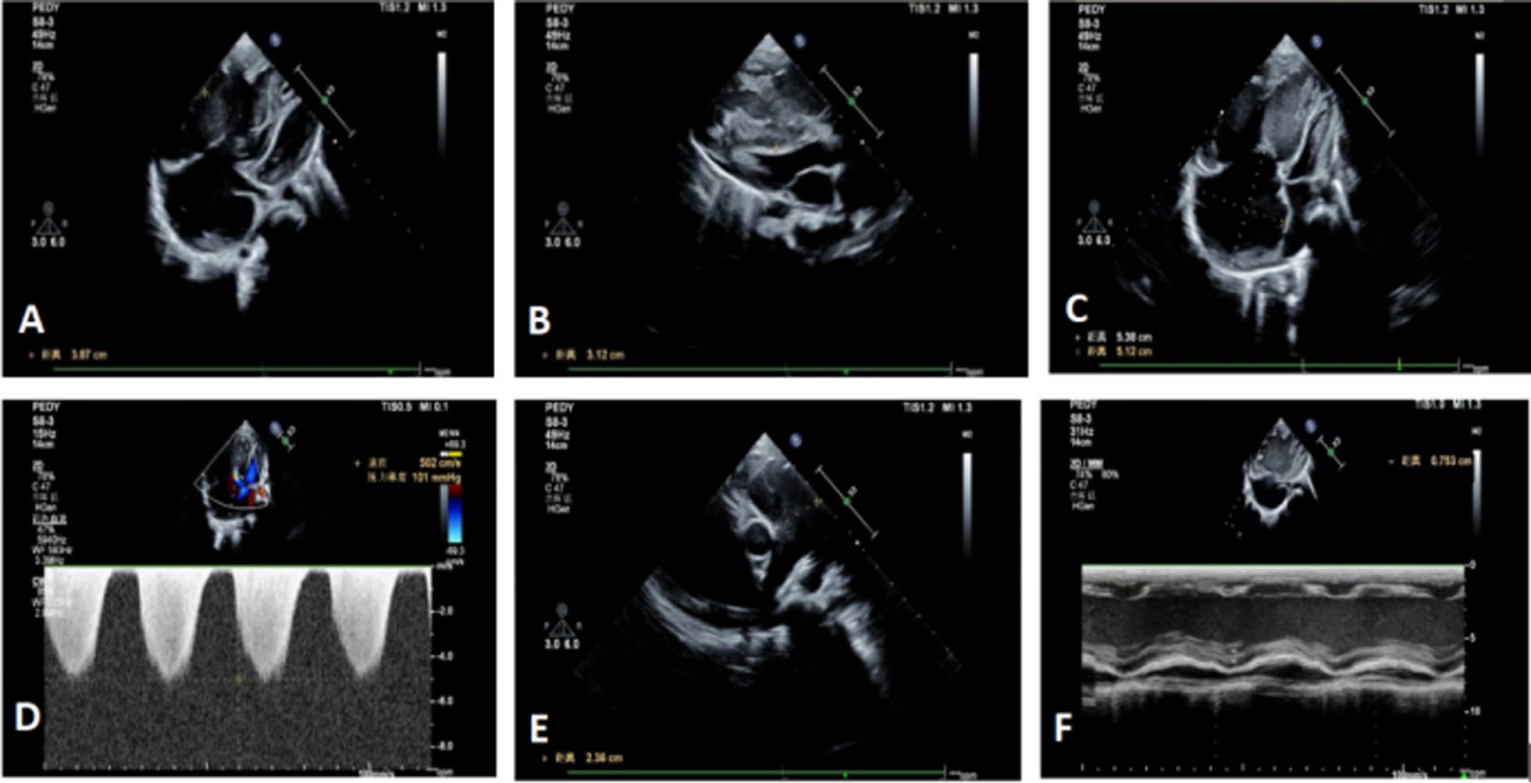
**a**, **b**, **c** More enlarged right ventricle and right atrium, right ventricle: 3.87 cm*3.12 cm(reference diameter 0.90 ~ 1.99 cm), right atrium: 5.38 cm*3.12 cm(reference diameter 2.19 cm ~ 2.41 cm). **d** Worsening tricuspid regurgitation (regurgitation velocity 5.02 m/s). **e** Widening pulmonary artery, main pulmonary artery diameter:2.36 cm(reference range:0.95 cm ~ 1.58 cm). **f** Significantly decreased right ventricular systolic function, tricuspid annular plane systolic excursion: 0.753 cm(reference range:1.25 cm ~ 1.88 cm)

Two months and five months after discharge, respectively, the child was re-admitted with pneumonia, worsening pulmonary hypertention and heart failure (Fig. 4). At last the boy died of heart failure six months after the initial discharge (Table 1). His parents refused to perform an autopsy.

**Table 1:**
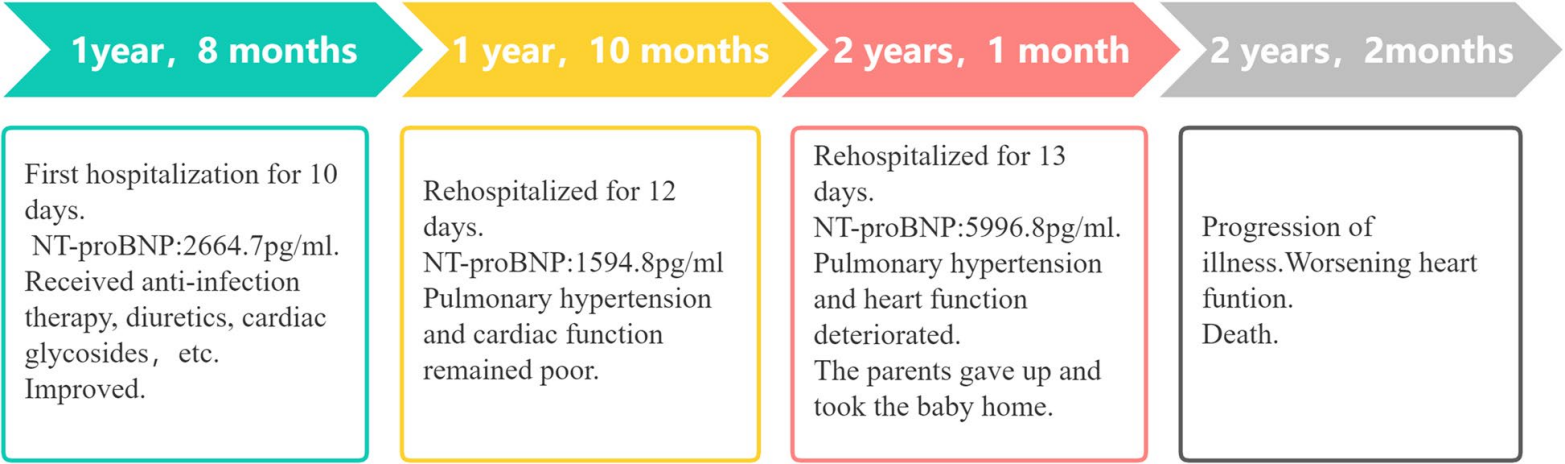
Time line of this case

## Discussion and conclusion

Noncompaction of ventricular myocardium (NVM) is an unclassified cardiomyopathy that was considered to be caused by premature termination of myocardial densification during embryonic development, with an incidence in adult is approximately 0.01% [1] and 0.14% in children [2]. NVM has three types: isolated left ventricular noncompaction (iLVNC), double ventricular involved and isolated right ventricular noncompaction (iRVNC), whose principle of classification is the lesion location. iRVNC is the most rare type [11], less than 30 cases in adults and 15 cases in children were reported. iRVNC doesn`t have specific diagnostic standard due to rarity and the abundance of trabecular muscle, and currently diagnostic criteria of iRVNC is formulated with reference to iLVNC: non-compact layer composed of abundant trabecular muscle and intertrabecular recesses and low-speed blood flow signal,non-compaction/compaction > 2.0 [11, 12]. In this case, the child showed decreased activity and enlarged liver. Imaging indicated the non-compact layer composed of abundant trabecular muscle and intertrabecular recesses and low-speed blood flow signal (non-compact layer/compact layer > 2.0), accompanied with increased right atrium and ventricle, left ventricular involvement was not obvious, so the diagnosis of this case is iRVNC. NVM could be associated with congenital heart disease, other primary cardiomyopathy or metabolic diseases. Most cases of iRVNC reported exist independently or combined with congenital heart disease [13–15]. And results of this case, for example, cardiac imaging, thyroid function and autoantibodies could exclude congenital heart disease, other primary cardiomyopathy and metabolic diseases.

NVM is an unclassified cardiomyopathy and some consider it genetically related, [3]. Currently, mutations of related genes, for example, some sarcomeric protein genes *MYH7*, *MYBPC3*, *JUP* and pathogenic genes of Barth syndrome *TAZ* could lead to NVM [5, 6]. The main mode of inheritance is autosomal dominant inheritance [5, 6], and NVM has not been reported due to *ACVRL1* mutation. *ACVRL1* is the pathogenic gene for hereditary hemorrhagic telangiectasia type 2 (HHT2). HHT is one sort of rare autosomal dominant disease with multiple arteriovenous malformations in skin, mucosa and common internal organs such as brain, lung, liver, nasal mucosa and gastrointestinal tract [7, 8], no other cardiac complications such as cardiomyopathy have been reported except pulmonary hypertension. *ACVRL1* gene is expressed on vascular endothelial cells resulting in abnormal TGF-β signal transduction pathway, which leads to arteriovenous fistula or proliferation of vascular wall, occlusion and reconstruction of pulmonary arterioles, and could eventually cause pulmonary hypertension [8–10]. But the boy, his mother and grandmother had no cyanosis, his CTA didn’t show any clumps, nodules, or cystomas that could be enhanced, nor did it show thickened, distorted, or prematurely imaged pulmonary veins, so there was no evidence for pulmonary arteriovenous fistula. In this case, *ACVRL1* heterozygous mutation from his mother was detected, both the child and his mother had recurrent epistaxis. Meanwhile, the child`s activity decreased and liver increased. Cardiac imaging suggested enlargement of right heart and moderate-severe tricuspid regurgitation, so iRVNC combined with HHT2 and pulmonary hypertension was confirmed. iRVNC can show complications such as right heart insufficiency, arrhythmia and pulmonary embolism [5, 15, 16], and pulmonary hypertension can be found in secondary pulmonary embolism [15, 16]. But the child's oxygen saturation was normal, pneumonia could be utilized to explain dyspnea, D-dimer and fibrinogen are within the normal range. No signs such as pulmonary artery filling defect were found in CTA, pulmonary embolism was less possible. Therefore, pulmonary hypertension was more likely to be caused by the other pulmonary vascular endothelial lesions except arteriovenous fistula related to the *ACVRL1* gene mutation.

NVM has been considered to be caused by stagnation in the process of embryonic cardiovascular development, in which the blood sinus between myocardial trabeculae is replaced by newly formed coronary artery and the loose reticular structure is gradually compacted [1], transforming growth factor-beta 1 (TGF-β1) signaling pathway plays an important role in myocardial smooth muscle cell differentiation, stromal formation and vascular differentiation [17], *ACVRL1* is a TGF-β1 receptor, which plays an important role in the properties of endothelial cells during angiogenesis [18].The mother of the child in this case also carried this gene mutation, cardiac ultrasound suggested the left ventricle apex noncompaction. Therefore, we speculate that *ACVRL1* may be one of the pathogenic genes of NVM. The abnormal protein conformation caused by the mutation of c.1450C > T:p.R484W may be the cause of this case.

However, according to the literature, 65.8% of the NVM patients have negative genetic testing results [19]. Recent data proposed additional etiopathogenic mechanisms, including acquired forms of LVNC secondary to overloading conditions [20–22]. The mechalism could be: 1.compensation for the failing ventricle to move physiologic stroke volumes, 2. dissection of the myocardium due to impaired gap junctions, 3. penetration of persisting sinusoids into the myocardial cavity, 4. a frustrate attempt of the failing left ventricular myocardium to hypertrophy, 5. enlargement of the endocardial surface to improve oxygenation from the ventricular side [20]. Since RVC is rare, there are no cases of acquired RVC for reference, we speculate that RVC could be secondary to right heart overloading conditions such as pulmonary hypertention, which is similar to LVNC. Furthermore, it is also unclear whether some other genetic changes might be causing RVC to appear in the same patient as HHT, and it’s just a coincidence.

As evidenced by the obtained results, we can conclude that: 1, *ACVRL1* may be the pathogenic gene of noncompaction and pulmonary hypertension in this case. 2, RVC may be secondary to pulmonary hypertention and right ventricular failure caused by *ACVRL1* mutation. 3, Noncompaction and *ACVRL1* mutation happened in the same case as a coincidence. To our knowledge, this is the first documented report linking *ACVRL1* mutation to NVM. Further studies of mechanisms of *ACVRL1* and NVM are thus to be confirmed.

## Supplementary Information

**Additional file 1.**

**Additional file 2.**

**Additional file 3.**

## Data Availability

All data generated during this study are included in this published article.and its supplementary information files.

## Abbreviations

NVM: Noncompaction of ventricular myocardium
iRVNC: Isolated right ventricular noncompaction
*ACVRL1*: Activin receptor-like kinase 1 gene
iLVNC: Isolated left ventricular noncompaction
HHT: Hereditary hemorrhagic telangiectasia
BP: Blood pressure
SPO_2_: Percutaneous oxygen saturation
hs-CRP: High-sensitivity C-reactive protein
CTA: Computed tomography angiography
TGF-β1: Transforming growth factor-beta 1
